# Undocumented immigrants and advanced heart failure therapies

**DOI:** 10.1016/j.jhlto.2025.100207

**Published:** 2025-01-07

**Authors:** Matthew Kogan, Sarah L. Kimball, Katherine Purrington, Jennifer Cedor, Omar K. Siddiqi

**Affiliations:** a Section of General Internal Medicine, Department of Medicine, Boston University Chobanian and Avedisian School of Medicine and Boston Medical Center; b Immigrant and Refugee Health Center, Boston Medical Center; c Health Law Advocates, Public Programs Advocacy and Immigrant Health Initiative; d Section of Cardiovascular Medicine, Department of Medicine, Boston University Chobanian and Avedisian School of Medicine and Boston Medical Center

**Keywords:** Transplant, Immigration, Social determinants of health

## Abstract

This study highlights the challenges undocumented immigrants face in accessing advanced heart failure therapies, despite contributing to the organ donor pool. Some key barriers are the lack of provider awareness, social support issues, and timing constraints. The survey conducted among cardiologists at Massachusetts teaching hospitals revealed that while most providers (82.5%) refer patients with undocumented status for advanced therapies, a significant percentage (87.5%) that still perceive immigration status as a barrier to transplantation. Addressing these misconceptions through legal partnerships and enhancing provider education is crucial for improving access to life-saving treatments for undocumented patients.

## Background

An estimated 10.5 – 12 million undocumented immigrants live in the United States. More marginalized populations in the US have higher rates of heart failure^1^ and the undocumented immigrant population is one of the least protected groups in our healthcare system. Since around 10% of patients with heart failure progress to advanced or end stage heart failure,^2^ there is a significant number of undocumented immigrants with advanced heart failure. Nationally, while 3% of organ donation comes from immigrants, they constitute 0.4% of all recipients.^3^ This is despite policy of the U.S. Organ Procurement Transplant Network (OPTN) that immigration status not be used as a factor in determining transplantation candidacy.^4^ This stark discrepancy underscores a profound inequity: despite their contributions to the organ donor pool, undocumented immigrants are underrepresented among organ recipients, highlighting a critical gap in our healthcare system's adherence to its own standards of inclusivity.

Undocumented immigrants face considerable barriers in accessing advanced heart failure therapies. Undocumented immigrants generally only qualify for Emergency Medicaid, which does not cover organ transplants. Some states have state-specific coverages which allow for expanded options for undocumented immigrants; however, this remains state dependent.

In addition, providers may hold misconceptions about immigrant patients’ level of social support. While there are occasionally patients in this population that are not candidates for heart transplantation, we are concerned that a significant percentage of undocumented immigrants are not referred for advanced therapies based on misconceptions rather than true barriers. Our investigation aims to discern how much of these misconceptions, or perceived barriers, stem from healthcare providers' lack of knowledge about laws related to Medicaid eligibility for advanced heart failure therapies.

## Methods

We distributed a survey to cardiologists at Massachusetts teaching hospitals to gauge their awareness of heart transplantation eligibility for undocumented immigrants. The majority of the teaching hospitals offer advanced therapies, and those that do not will transfer those patients to nearby hospitals when in need. The survey, consisting of yes/no and open-response questions, targeted providers' understanding of what options are available to undocumented immigrants in end-stage heart failure. The survey was distributed via email sent out by program directors across the cardiology training programs in Massachusetts.

### Survey results

**Fig. 1:**
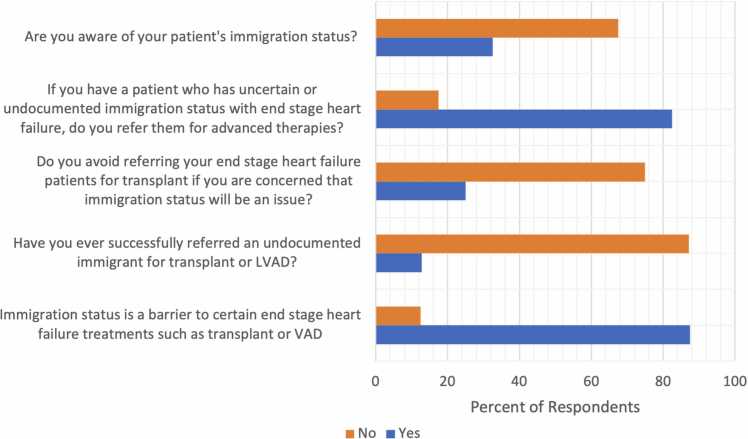
Survey questions that were administered to cardiologists in Massachusetts and corresponding responses.

There were 40 survey respondents. Among the respondents, 27.5% were general cardiology fellows, 2.5% were advanced cardiology fellows, 17.5% were advanced heart failure and transplant attendings, 45% were non-heart failure cardiology attendings, and 7.5% were cardiology APPs. Of the respondents, 32.5% were aware of their patients’ immigration status, 82.5% refer patients with undocumented immigration status for advanced therapies, while 25% say they avoid referring for transplant if they are concerned immigration status will be an issue. 12.82% of the respondents have successfully referred an undocumented immigrant for transplant or LVAD and 87.5% of respondents say that immigration status is a barrier to transplant (Figure 1).

## Discussion

We investigated providers' perceptions of immigration status as a barrier to heart transplantation. Most providers (82.5%) refer patients with undocumented immigration status for advanced therapies, however, a significant proportion (87.5%) also perceives immigration status as a barrier.

Survey responses revealed three primary barriers: immigration legal hurdles, social support issues, and timing constraints. Many providers' limited grasp of immigration law and its impact on public benefits led to perceived barriers. For instance, 25% of respondents avoided transplant referrals due to immigration status.

In many situations, patients have options to overcome legal barriers, or the legal barrier that the provider perceives does not actually exist. In several states, PRUCOL individuals can enroll in comprehensive Medicaid coverage. Instead of equating undocumented immigration status with transplant ineligibility, healthcare systems, acting through Medico Legal Partnership (MLP) models should explore whether patients already have qualifying immigration documents or could obtain such documentation by working with an attorney.

Respondents highlighted social barriers as a primary reason for undocumented immigrants’ limited access to heart transplantation. However, when assessing the level of social support, it is important to underscore that, with adequate insurance coverage, patients may be eligible for patient care assistants, home nurses, and transportation to appointments.

The role of adequate social support in predicting successful heart transplant outcomes and the use of social support in establishing transplant eligibility is controversial. OPTN policy states that there is no association between social support and overall or graft survival; however, there is limited data that associates social support with improved quality of life post-transplant.^4^ Additionally, there is likely bias in assessing levels of social support with immigrant populations since they are more likely to have limited English proficiency or inflexible employment schedules.^4^ Per OPTN, the level of social support that a patient has should not affect “access to life-saving care.”^5^ This underscores the danger of assumptions about social barriers, potentially exacerbating inequities rather than addressing them, especially when state resources and support mechanisms can provide significant assistance to mitigate social barriers.

Our study limitations include surveying providers on general perceptions, which lacks specificity regarding reasons for referral avoidance and outcomes of legal immigration consultation. These limitations underscore the need for future research to delve deeper into these aspects. Our study is also limited in sample size given; however, this is a sample from a population of cardiologists in hospitals which often see a high immigrant population and in a state which offers many insurance options for undocumented immigrants. Therefore, despite the small sample size, it is reasonable to conclude that the actual levels of provider awareness would be lower on a national scale, but additional larger surveys would be needed to provide definitive proof.

Our survey highlights the importance of provider awareness and early referrals to immigration legal teams. Prioritizing these referrals can mitigate timing issues. Raising provider awareness is essential to reduce instances of patients not being referred for transplant, emphasizing equitable access to life-saving treatments for undocumented immigrants with end-stage heart failure.

## Declaration of Competing Interest

The authors declare that they have no known competing financial interests or personal relationships that could have appeared to influence the work reported in this paper.
